# Albumin levels predict mortality in sepsis patients with acute kidney injury undergoing continuous renal replacement therapy: a secondary analysis based on a retrospective cohort study

**DOI:** 10.1186/s12882-021-02629-y

**Published:** 2022-02-02

**Authors:** Song Sheng, Yan-Hong Zhang, Hang-Kun Ma, Ye Huang

**Affiliations:** Emergency Department, China Academy of Chinese Medical Science Xiyuan Hospital, Beijing, 100091 China

## Abstract

**Background:**

Albumin (ALB) levels are negatively associated with mortality in patients with sepsis. However, among sepsis patients with acute kidney injury (AKI) undergoing continuous renal replacement therapy (CRRT), there has been no similar study on the correlation between ALB levels and mortality alone. This study tested the hypothesis that ALB levels are negatively associated with mortality among such patients.

**Methods:**

We conducted a secondary analysis of 794 septic patients who were diagnosed with AKI and underwent CRRT in South Korea. For the Kaplan–Meier survival analysis, Cox proportional hazards models were used to study the hypotheses, with adjustments for the pertinent covariables. We also explore the possible nonlinear relationship and conducted sensitivity analyses including subgroup analyses and tests for interactions to investigate the association further. Additionally, ALB was used to construct model and we then compared the performance of ALB with that of APACHE II and SOFA in predicting mortality.

**Results:**

The ALB level was an independent prognostic factor for death at 28 and 90 days after CRRT initiation (*HR* = 0.75, 95% CI: 0.62–0.90, *P* = 0.0024 for death at 28 days and *HR* = 0.73, 95% CI: 0.63–0.86, *P* < 0.0001 for death at 90 days). A nonlinear association was not identified between ALB levels and the endpoints. Subgroup analyses and tests for interactions indicated that HCO_3_ and CRP played an interactive role in the association. ROC analysis indicated ALB, SOFA and APACHE-II were separately inadequate for clinical applications.

**Conclusion:**

A 1 g/dL increase in ALB levels was independently associated with a 25 and 27% decrease in the risk of death at 28 and 90 days, respectively. However, this conclusion needs to be taken with caution as this study has several limitations.

**Supplementary Information:**

The online version contains supplementary material available at 10.1186/s12882-021-02629-y.

## Introduction

Sepsis is a global healthcare issue that continues to be the leading cause of mortality from infection. It is a systemic inflammatory response that usually progresses to multiple organ dysfunction syndrome, especially acute kidney injury (AKI) [1]. For those critically ill among sepsis patients with AKI, continuous renal replacement therapy (CRRT) is a broadly-accepted treatment to optimize fluid and electrolyte management. Despite the active application of CRRT, a previous study revealed that sepsis patients with AKI undergoing CRRT had substantially higher mortality than sepsis patients (over 60% vs over 30%) [2–4]. Therefore, the early recognition and the identification of prognostic factors for mortality in such high-risk groups are urgently required to prevent death and improve prognosis.

The albumin (ALB) level is a common laboratory indicator that is negatively associated with mortality in patients with sepsis [5]. It is well-known that hypoproteinemia is a widespread clinical complication in patients with sepsis, and that the ALB level is an early predictor for mortality risk among such patients [6, 7]. However, there has been no similar study on the correlation between ALB levels and death alone in sepsis patients with AKI undergoing CRRT. At present, the extent of the negative association that ALB levels may have and whether it will provide additional prognostic information in these patients is unclear. Therefore, our study aimed to analyze the association between ALB levels and mortality in sepsis patients with AKI undergoing CRRT.

## Methods

### Study population

Our study was a secondary analysis based on a retrospective cohort study. The existing data were obtained from DATADRYAD (https://datadryad.org/stash), a community-owned resource where raw clinical data may be acquired freely because all copyrights of the uploaded data have been waived. According to the Terms of Service, we cited the data package Jung, Su-Young J et al. (2019), Data from: Phosphate is a potential biomarker of disease severity and predicts adverse outcomes in acute kidney injury patients undergoing continuous renal replacement therapy, Dryad, Dataset, 10.5061/dryad.6v0j9 [3].

**Fig. 1:**
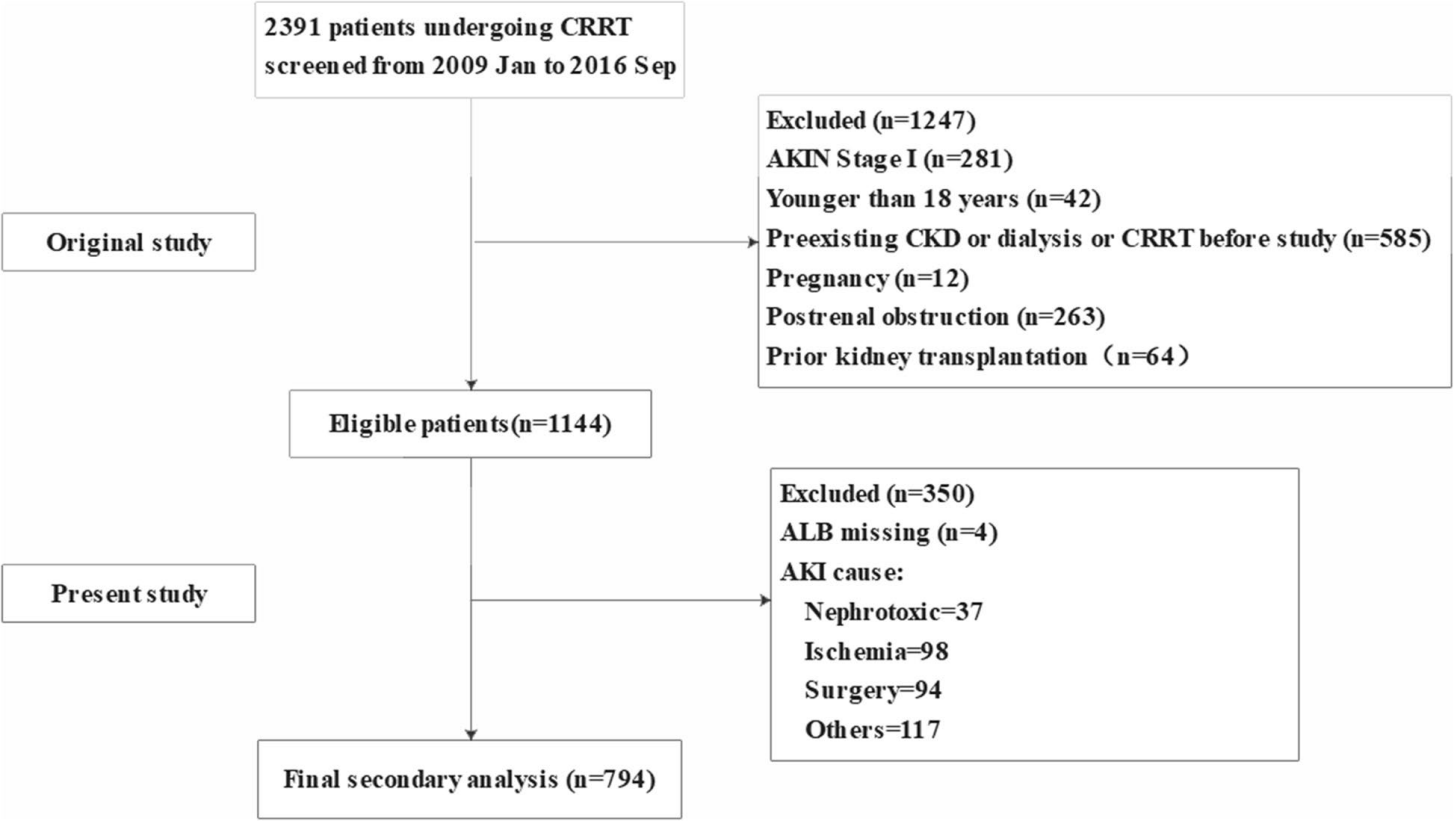
The flowchart of patient selection in this study

In their research, data were obtained from the medical records of 2391 patients who received CRRT in the intensive care units of Yonsei University Health System Severance Hospital and National Health Insurance Service Medical Center Ilsan hospital in South Korea between January 2009 and September 2016. Among the 2391 patients, those classified as stage II or above according to Acute Kidney Injury Network (AKIN) criteria (> 2-fold increase in serum creatinine or urine output [UO] < 0.5 mL∙kg^− 1^∙h^− 1^ for 12 h) were eligible [8]. The exclusion criteria of the original study were as follows: age < 18 years, pregnancy or lactation, history of chronic kidney disease (CKD) or of dialysis or CRRT before the study, postrenal obstruction, and prior kidney transplantation. In the present study, sepsis patients without missing ALB variables were retained. Sepsis was identified according to the 2001 SCCM/ESICM/ACCP/ATS/SIS International Sepsis Definitions Conference guidelines [9]. Thus, 794 patients were included in the analysis. The flowchart of patient selection is presented in Fig. 1.

The original study was retrospectively approved by the Yonsei University Health System Severance Hospital Institutional Review Board (No. 4–2016-1073). The requirement of the informed consent was waived because of the retrospective nature of the study [10].

### Data collection

Demographic and clinical data, including age, sex, body mass index (BMI), systolic blood pressure (SBP), diastolic blood pressure (DBP), mean arterial pressure (MAP), age-adjusted Charlson comorbidity index (aCCI) [11], and mechanical ventilation (MV) at CRRT initiation, were collected before starting CRRT (0 h). Jung et al. also recorded biochemical laboratory data at CRRT initiation, including data on hemoglobin (HGB), white blood cell count (WBC), C-reactive protein (CRP), serum creatinine (Cr), blood urea nitrogen (BUN), glomerular filtration rate (GFR), ALB, bicarbonate (HCO_3_), potassium (K), and phosphate (P). To evaluate disease severity and organ failure, the AKIN stage, Sequential Organ Failure Assessment (SOFA) score [12], and Acute Physiology and Chronic Health Evaluation II (APACHE II) score [13] were also recorded at CRRT initiation.

Upon the development of AKI in the patients with sepsis, nephrologists decided whether and when to initiate CRRT in those who were critically ill. General indications included sustained oliguria, uncontrolled volume overload, intractable hyperkalemia, or metabolic acidosis. The CRRT protocol was specifically described in the original study [3]. Jung et al. also collected 2 h UO and total effluent volume after CRRT initiation.

### Study endpoints

The study endpoints were death at 28 days and 90 days after CRRT initiation.

### Statistical analysis

The ALB levels were divided into tertiles. Data were expressed as means ± SDs (normal distribution) or medians (Q1**–**Q3) (abnormal distribution) for continuous variables, and as frequencies and percentages for the categorical variables. The effect of the ALB level tertiles on the endpoints were evaluated using Kaplan–Meier (K**–**M) curves and log-rank tests. Hazard ratios (HR) and 95% confidence intervals (CI) for death at 28 days and 90 days after CRRT initiation, negatively correlated with ALB levels, were estimated using the Cox proportional hazards regression model. Based on the STROBE statement [14], we displayed the results of multiple models, including non-adjusted, multivariate adjusted (adjusted I and adjusted II), and fully-adjusted models. Covariates were included as potential confounders in the adjusted I model if they changed the HRs of the ALB levels at the endpoints by more than 10%. In the adjusted II model, we included confounders that changed the HRs by more than 10% or were significantly associated with the endpoints (*P* < 0.1). We then performed linear trend tests by entering the median value of each category of ALB level as a continuous variable in the four models [15]. We used multiple imputations (MI) based on five replications and the chained equation approach to account for missing data for K, HCO_3_, P, BMI, SBP, DBP, MV, WBC, HGB, BUN, Cr, CRP, GFR, UO, APACHE-II score, and SOFA score [16]. The HRs, 95% CIs, and *P* values of multiple Cox regression of the five replications were combined according to Rubin’s rule. We also explored whether there was a possible nonlinear relationship between the ALB level and the endpoints using the generalized additive model (GAM) and smooth curve fitting. If such a relationship was observed, a two-piecewise linear regression model was used to determine the threshold effect of ALB levels on the endpoints [17]. When the threshold value of the ALB level, at which the correlation between the ALB level and the endpoints became significant in the smoothed curve, the recurrence method was utilized to detect the inflection point that gave the maximum model likelihood [18]. Moreover, the bootstrap resampling method (1000 times) was used to determine the 95% CI of the threshold. Interaction and subgroup analyses were conducted according to age (< 60 and ≥ 60 years), sex, K (< 3.5 and ≥ 3.5 mmol/L; < 5.5, ≥ 5.5 mmol/L), HCO_3_ (< 22 and ≥ 22 mmol/L), P (< 4.3 and ≥ 4.3 mg/dL), aCCI score (dichotomy), BMI (< 18.5 and ≥ 18.5 kg/m^2^; < 23.9, and ≥ 23.9 kg/m^2^), SBP (< 90 and ≥ 90 mmHg), DBP (< 60 and ≥ 60 mmHg), MAP (< 70 and ≥ 70 mmHg), MV, WBC (< 4000 and ≥ 4000/μL), HGB (< 12 and ≥ 12 g/dL), BUN (tertile), Cr (tertile), CRP (tertile), GFR (tertile), UO (tertile), APACHE-II score (dichotomy), SOFA score (dichotomy), Indications for CRRT, and AKIN stage. In the adjusted II model, each stratification was adjusted for all variables aside from the stratification variable itself [19]. Additionally, we explored the potential unmeasured confounding between the ALB level and the endpoints using an E-value calculator (https://mmathur.shinyapps.io/evalue/) [20]. The E-value quantifies the magnitude of an unmeasured confounder that could negate the observed correlation between the ALB levels and the endpoints [21]. Finally, receiver-operating characteristic (ROC) curve analysis was performed, and areas under the curve (AUC), accuracy, sensitivity, specificity and Youden index were reported to evaluate the performance of ALB, SOFA and APACHE-II predicting the death at 28 days and 90 days, respectively. DeLong’s test was applied for pairwise comparisons between the AUCs. All the mentioned analyses except the E-value computation were conducted using R 4.0.3 (http://www.R-project.org). All the probabilities were two tailed, and *P* < 0.05 was considered to be statistically significant.

## Results

### Baseline characteristics

The mean age of the patients was 63.53 ± 14.19 years, and 61.90% of them were male. The mortality rates at 28 days and 90 days were 62.46 and 73.43%, respectively. The mean times to death at 28 days and 90 days were 18.35 ± 11.62 days and 30.04 ± 37.48 days, respectively. There were no significant differences in age, K, P, aCCI, BMI, SBP, DBP, MAP, WBC, BUN, Cr, GFR, UO, SOFA score, CRRT dose (total effluent volume), sex, indications for CRRT, and AKIN stage among patients in the ALB tertile groups. Compared with the patients in the T1 group, the rest had significantly longer survival times, higher HCO_3_ and HGB, and lower MV percentage, CRP, and APACHE-II score. The baseline characteristics of the patients are shown in Table 1.

**Table 1:** Baseline characteristics of the patients

ALB tertiles	TI group (0.00–2.20 g/dl)	T2 group (2.30–2.70 g/dl)	T3 group (2.80–5.90 g/dl)	***P*** value
**N (794)**	218	286	290	
**ALB**	1.92 ± 0.32	2.50 ± 0.14	3.17 ± 0.39	< 0.001
**Time to death = 28 days**	28.00 (3.00–28.00)	28.00 (4.00–28.00)	28.00 (7.00–28.00)	0.024
**Time to death = 90 days**	3.00 (1.00–29.69)	5.80 (1.00–58.96)	14.40 (1.00–90.00)	< 0.001
**Age (years)**	65.00 ± 12.85	63.72 ± 14.49	62.49 ± 14.59	0.137
**K (mmol/L)**	4.76 ± 1.15	4.59 ± 0.99	4.77 ± 1.12	0.098
**HCO**_**3**_ **(mmol/L)**	15.71 ± 5.18	17.10 ± 5.67	17.73 ± 5.76	< 0.001
**P (mg/dL)**	5.53 ± 2.26	5.47 ± 2.15	5.90 ± 2.45	0.067
**aCCI score**	2.00 (1.00–5.00)	3.00 (2.00–5.00)	3.00 (1.25–4.00)	0.155
**BMI (kg/m**^**2**^**)**	23.36 ± 4.48	23.39 ± 4.34	23.89 ± 4.54	0.309
**SBP (mmHg)**	110.44 ± 20.62	112.21 ± 21.71	111.71 ± 20.50	0.632
**DBP (mmHg)**	61.00 ± 13.91	61.05 ± 14.70	60.28 ± 13.79	0.774
**MAP (mmHg)**	77.42 ± 14.02	77.76 ± 15.58	77.23 ± 14.62	0.909
**WBC (uL)**	10,690.00 (5130.00–18,960.00)	11,070.00 (6355.00–18,285.00)	12,060.00 (8170.00–18,740.00)	0.171
**HGB (g/dL)**	9.23 ± 2.06	9.73 ± 1.98	10.03 ± 2.37	< 0.001
**BUN (mg/dL)**	55.00 (36.00–78.00)	49.00 (35.00–74.00)	48.00 (34.00–71.00)	0.162
**Cr (mg/dL)**	2.26 (1.59–3.16)	2.42 (1.67–3.39)	2.34 (1.66–3.36)	0.358
**CRP (mg/L)**	89.60 (20.10–199.50)	73.25 (20.55–176.70)	46.90 (15.00–133.50)	0.011
**GFR (ml/min/1.73 m**^**2**^**)**	27.05 (18.27–39.55)	26.45 (17.62–38.98)	26.30 (16.17–38.60)	0.547
**UO (mL)**	25.00 (4.25–100.00)	30.00 (5.00–96.25)	40.00 (10.00–100.00)	0.139
**APACHE-II score**	28.37 ± 7.63	27.46 ± 7.64	26.60 ± 8.48	0.047
**SOFA score**	12.26 ± 3.38	12.16 ± 3.45	11.64 ± 3.73	0.09
**CRRT dose (mL/kg)**	36.99 ± 5.01	36.62 ± 4.85	36.57 ± 4.39	0.579
**Sex**				0.187
**Male**	132 (60.55%)	189 (66.08%)	171 (58.97%)	
**Female**	86 (39.45%)	97 (33.92%)	119 (41.03%)	
**MV**				0.049
**No**	36 (16.51%)	57 (19.93%)	73 (25.26%)	
**Yes**	182 (83.49%)	229 (80.07%)	216 (74.74%)	
**Indications for CRRT**				0.326
**Volume overload**	22 (10.09%)	35 (12.24%)	45 (15.52%)	
**metabolic acidosis**	49 (22.48%)	73 (25.52%)	61 (21.03%)	
**hyperkalemia**	14 (6.42%)	8 (2.80%)	17 (5.86%)	
**uremia**	23 (10.55%)	33 (11.54%)	25 (8.62%)	
**oliguria**	53 (24.31%)	77 (26.92%)	75 (25.86%)	
**others**	57 (26.15%)	60 (20.98%)	67 (23.10%)	
**AKIN stage**				0.983
**stage II**	59 (27.06%)	76 (26.57%)	79 (27.24%)	
**stage III**	159 (72.94%)	210 (73.43%)	211 (72.76%)	

### Univariate analysis between ALB levels and the endpoints

The results of the univariate analyses are presented in Table S1. P, aCCI, BMI, SBP, DBP, MAP, MV, Cr, ALB levels, UO, APACHE-II score, SOFA score, and CRRT were associated with death at both 28 and 90 days (*P* < 0.05). GFR was only correlated with death at 90 days (*P* < 0.05).

### Kaplan–Meier curves of survival probability

**Fig. 2:**
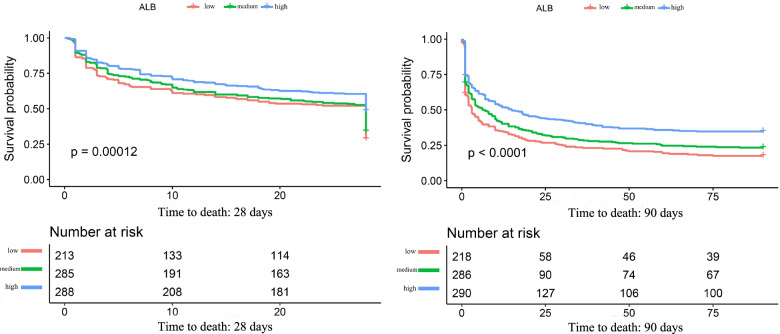
K**–**M curves of the survival probabilities of ALB tertiles at 28 days and 90 days

The K**–**M curves of the survival probabilities of the ALB tertiles are shown in Fig. 2. From the chart, we clearly see that the survival probabilities among ALB tertiles at 28 days and 90 days were significantly different (log-rank test *P* = 0.00012 for 28 days and *P* < 0.0001 for 90 days, respectively).

### Multivariate analysis between ALB levels and the endpoints

In the crude model, the ALB level was negatively correlated with death at 28 days and 90 days (*HR* = 0.71, 95% CI: 0.61–0.82, *P* < 0.0001 for 28 days; *HR* = 0.70, 95% CI: 0.61–0.80, *P* < 0.0001 for 90 days). In the adjusted I and II models, the HRs of the negative association were listed as follows: *HR* = 0.74, 95% CI: 0.63–0.88, *P* = 0.0004 and *HR* = 0.75, 95% CI: 0.62–0.90, *P* < 0.0001, respectively for death at 28 days; *HR* = 0.75, 95% CI: 0.65–0.87, *P* = 0.0001 and *HR* = 0.73, 95% CI: 0.63–0.86, *P* < 0.0001, respectively for death at 90 days. In the fully adjusted model, the ALB level was also negatively related with the endpoints (*HR* = 0.72, 95% CI: 0.58–0.90, *P* = 0.0039 for death at 28 days; *HR* = 0.68, 95% CI: 0.55–0.85, *P* = 0.0005 for death at 90 days). The results were shown in Table 2.

**Table 2:** Results of the multivariate analysis between ALB and the endpoints

N (death at 28 days)	794	757	581	482
**Models**	Non-adjusted HR (95% CI) P value	Adjusted I HR (95% CI) P value	Adjusted II HR (95% CI) P value	Fully adjusted HR (95% CI) P value
**ALB (g/dL)**	0.71 (0.61–0.82) < 0.0001	0.74 (0.63–0.88) 0.0004	0.75 (0.62–0.90) 0.0024	0.72 (0.58–0.90) 0.0039
**ALB tertiles**				
**Low (0.00–2.20 g/dl)**	Ref	Ref	Ref	Ref
**Middle (2.30–2.70 g/dl)**	0.87 (0.71–1.08) 0.2042	0.86 (0.69–1.08) 0.1944	0.75 (0.57–0.98) 0.0332	0.81 (0.60–1.09) 0.1700
**Middle (2.80–5.90 g/dl)**	0.63 (0.51–0.79) < 0.0001	0.67 (0.53–0.85) 0.0008	0.64 (0.49–0.85) 0.0019	0.65 (0.47–0.89) 0.0069
**P for trend**	< 0.0001	0.0006	0.0022	0.0069
**N (death at 90 days)**	794	790	731	482
**Models**	Non-adjusted HR (95% CI) P value	Adjusted I HR (95% CI) *P* value	Adjusted II HR (95% CI) P value	Fully adjusted HR (95% CI) P value
**ALB (g/dL)**	0.70 (0.61–0.80) < 0.0001	0.75 (0.65–0.87) 0.0001	0.73 (0.63–0.86) < 0.0001	0.68 (0.55–0.85) 0.0005
**ALB tertiles**				
**low**	Ref	Ref	Ref	Ref
**middle**	0.83 (0.68–1.02) 0.0713	0.83 (0.68–1.02) 0.0762	0.81 (0.65–1.00) 0.0547	0.72 (0.55–0.96) 0.0253
**high**	0.62 (0.50–0.76) < 0.0001	0.67 (0.54–0.82) 0.0002	0.63 (0.50–0.79) < 0.0001	0.60 (0.45–0.80) 0.0005
**P for trend**	< 0.0001	0.0001	< 0.0001	0.0006

Non-adjusted model adjusted for: None

Adjusted I model (death at 28 days) adjusted for: age, sex, P, BUN, Cr, UO, SOFA score, and Indications for CRRT

Adjusted I model (death at 90 days) adjusted for: age, sex, BUN, Cr, and SOFA scores

Adjusted II model (death at 28 days) adjusted for: age, sex, K, HCO3, P, aCCI, BMI, SBP, DBP, MAP, MV, WBC, HGB, BUN, Cr, CRP, UO, APACHE-II score, SOFA score, Indications for CRRT, CRRT dose, and AKIN

Adjusted II model (death at 90 days) adjusted for: age, sex, K, HCO3, P, aCCI, BMI, SBP, DBP, MAP, MV, WBC, HGB, BUN, Cr, GFR, UO, APACHE-II score, SOFA score, Indications for CRRT, CRRT dose, and AKIN

Fully adjusted model adjusted for: all variables except ALB and the endpoints

In the sensitivity analysis, we also viewed the ALB level as a categorical variable (tertile), and the same trends were detected in the four models (*P* for trend < 0.05). We found that some variables for K, HCO3, P, BMI, SBP, DBP, MV, WBC, HGB, BUN, Cr, CRP, GFR, UO, APACHE II score, and SOFA score were missing in the raw data, with the numbers of patients with missing variables being 5, 110, 30, 16, 2, 2, 1, 5, 1, 2, 1, 166, 2, 5, 12, and 3, respectively. The results of the MI indicated that between the raw data and combined imputed data, there was only a slight difference in HR (Table S2). In other words, we concluded that the data were missing at random, which would not significantly affect the results of the analysis in the four models.

### Linearity or non-linearity of the correlation between ALB levels and the endpoints

Through the application of smooth curve fitting, we determined that the associations between the ALB levels and death at 28 days and 90 days were nonlinear after adjusting for variables in the adjusted I and II models (Fig. S1). By calculation and bootstrap resampling, the inflection points for death at 28 days were found to be 2.20 g/dl (95% CI: 2.10–2.24) and 2.10 g/dl (95% CI: 1.80–2.21) after adjusting variables in adjusted I and adjusted II models, respectively. For death at 90 days, the thresholds were 1.84 g/dl (95% CI: 1.79–2.01) and 1.80 g/dl (95% CI: 1.71–2.04) after adjusting variables in the two models. However, the log-likelihood ratio test indicated that *P* values were less than 0.05 for death at both 28 days and 90 days (Table S3). As a result, the correlation between the ALB levels and the endpoints was linear.

### The results of subgroup analysis and test for interaction

**Fig. 3:**
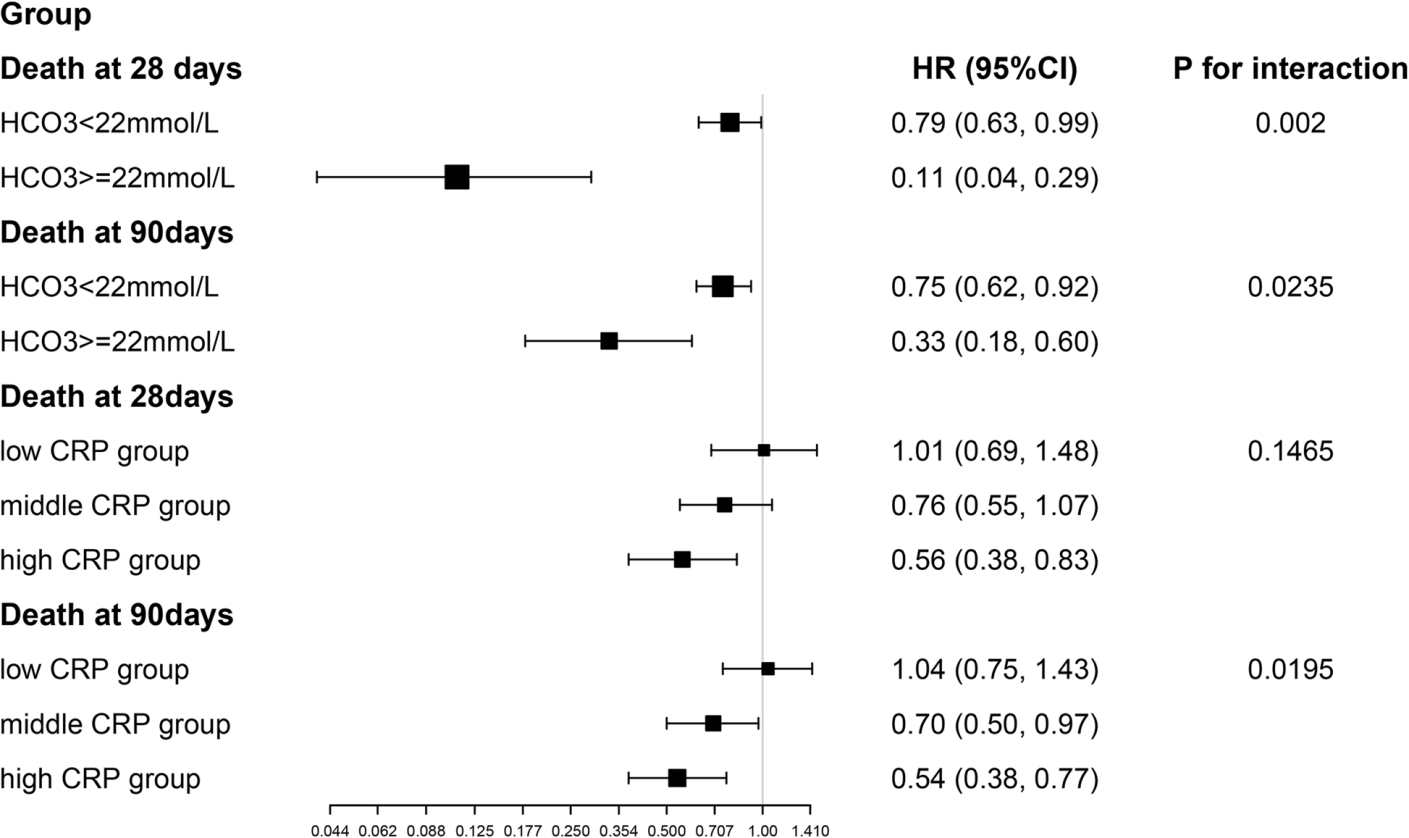
Results of the test for interaction in the HCO_3_ and CRP subgroups

The subgroup analyses and tests for the interaction of the correlations between ALB levels and death at 28 days and 90 days are presented in Table S4. The negative correlations between ALB levels and the endpoints were stable in nearly all subgroups. The interaction analysis revealed that HCO_3_ and CRP played an interactive role in the association between ALB levels and mortality (Fig. 3). The patients with HCO_3_ ≥ 22 mmol/L had lower HRs (*HR* = 0.11, 95% CI: 0.04–0.29 for death at 28 days; *HR* = 0.33, 95% CI: 0.18–0.60 for death at 90 days) than those with HCO_3_ < 22 mmol/L (*HR* = 0.79, 95% CI: 0.63–0.99, *P* for interaction = 0.0020 for death at 28 days; *HR* = 0.75, 95% CI: 0.62–0.92, *P* for interaction = 0.0235 for death at 90 days). In addition, the HR between ALB levels and death at 90 days was significantly lower in patients with high CRP (low CRP group: *HR* = 1.04, 95% CI: 0.75–1.43; middle CRP group: *HR* = 0.70, 95% CI: 0.50–0.97; high CRP group: *HR* = 0.64, 95% CI: 0.38–0.77; *P* for interaction = 0.0195). The same trend was also found between ALB levels and death at 28 days among the CRP subgroups, but the difference was not statistically significant (*P* for interaction = 0.1465).

### The ROC analysis and AUCs for ALB, SOFA and APACHE-II predicting the death

**Fig. 4:**
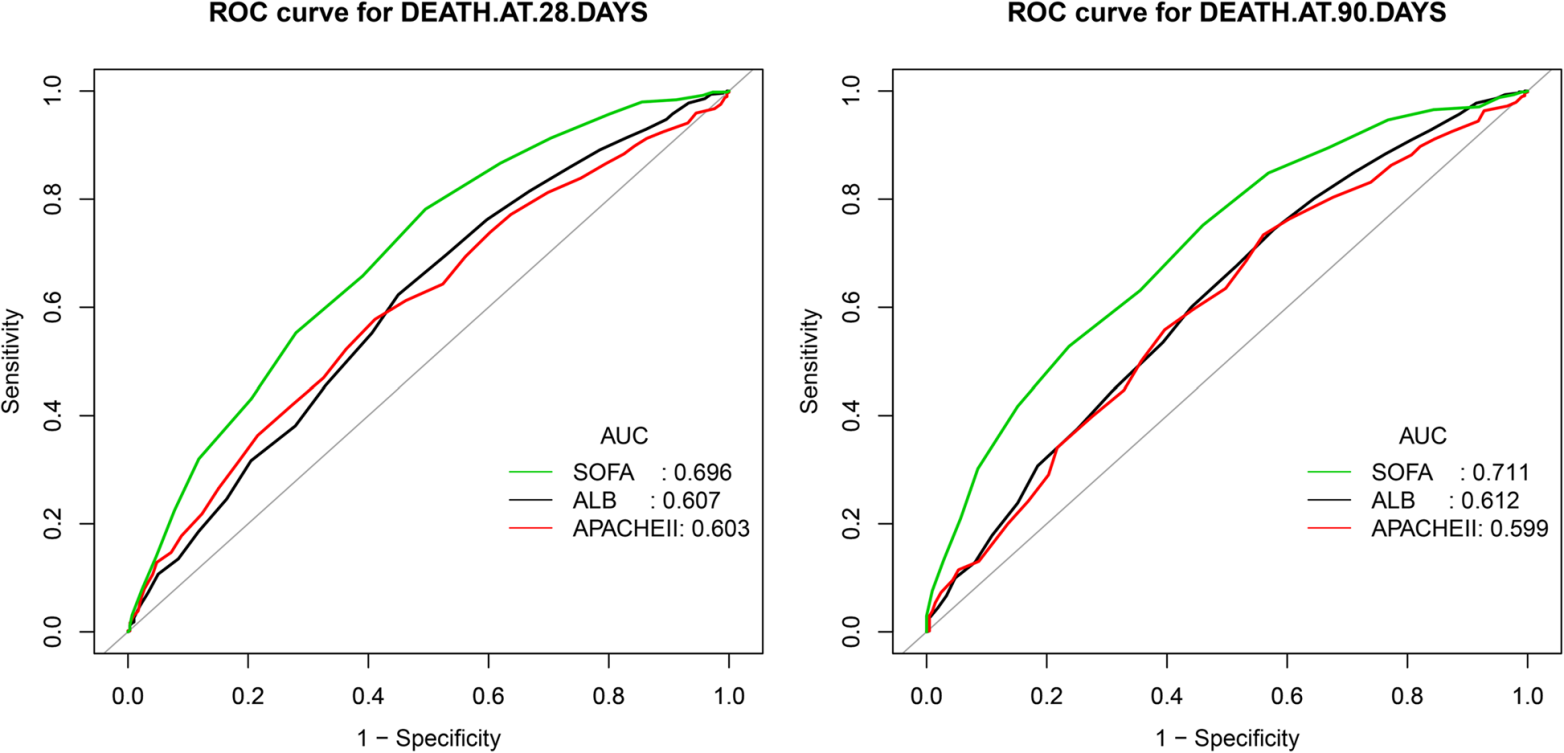
ROC curves of ALB, SOFA and APACHE-II predicting the death at 28 days and 90 days

We observed that the ALB performs comparably to the APACHE-II model in predicting the death at 28 days and 90 days (DeLong’s test *P* = 0.8741 for death at 28 days; DeLong’s test *P* = 0.7977 for death at 90 days). When compared to ALB and APACHE-II, SOFA enhanced the performance to predict the death at 28 days (SOFA vs ALB: DeLong’s test *P* = 0.0013, SOFA vs APACHE-II: DeLong’s test *P* < 0.0001) and 90 days (SOFA vs ALB: DeLong’s test *P* = 0.0010, SOFA vs APACHE-II: DeLong’s test *P* = 0.0001) with higher AUC. The results of ROC analysis and AUCs of ALB, SOFA and APACHE-II are shown in Fig. 4 and Table 3.

**Table 3:** Results of the ROC analysis

death at 28 days	AUC	95%CI low	95%CI up	Specificity	Sensitivity	Accuracy	Youden
SOFA	0.6959	0.6581	0.7336	0.5051	0.7814	0.6776	0.2865
ALB	0.6067	0.5660	0.6474	0.5503	0.6230	0.5957	0.1733
APAII	0.6030	0.5627	0.6434	0.5890	0.5776	0.5818	0.1666
**death at 90 days**	**AUC**	**95%CI low**	**95%CI up**	**Specificity**	**Sensitivity**	**Accuracy**	**Youden**
SOFA	0.7106	0.6706	0.7506	0.5403	0.7517	0.6953	0.2920
ALB	0.6119	0.5670	0.6569	0.4218	0.7444	0.6587	0.1662
APAII	0.5991	0.5540	0.6442	0.4396	0.7339	0.6560	0.1735

## Discussion

In our study, the ALB level was identified as a significant and independent prognostic factor for death at 28 days and 90 days after CRRT initiation among sepsis patients with AKI undergoing CRRT. The patients with a high ALB level had a lower risk of death at 28 days and 90 days than those with a low ALB level after adjusting for variables in the adjust II model (*P* for trend = 0.022 for death at 28 days; *P* for trend < 0.0001 for death at 90 days). Indeed, a 1 g/dL increase in the ALB levels was independently associated with a 25 and 27% lower risk of death at 28 days and 90 days, respectively (*HR* = 0.75, 95% CI: 0.62–0.90 for death at 28 days; *HR* = 0.73, 95% CI: 0.63–0.79 for death at 90 days), further confirming the negative relationship between ALB levels and death. Furthermore, we found a linear rather than a curvilinear correlation between ALB levels and the endpoints (log likelihood ratio test *P* > 0.05). In addition, we found that both HCO_3_ and CRP levels before CRRT initiation influenced this correlation, and that patients with HCO3 (≥ 22 mmol/L) and high CRP benefited more from elevated level of ALB. High CRP levels reflect severe tissue injury and infection which lead to remarkable hypoproteinemia [22]. Consequently, the benefit of albumin supplementation is especially evident in high CRP group. Also, the patients with HCO3 ≥ 22 mmol/L had lower HRs than those with HCO3 < 22 mmol/L. I think it has implications for clinicians: acidosis must be rectified before starting albumin infusion or the probable benefit of albumin infusion is limited in these patients. ALB was independently negatively correlated with mortality in sepsis patients with AKI undergoing CRRT. However, ROC analysis indicated whether ALB or SOFA and APACHE-II were separately inadequate for clinical applications due to low sensitivity, specificity, accuracy and AUC. There is a need for a better predictive indicator which can overcome the limitations of current predictive scores and risk factors.

In a multicenter retrospective observational study, Kawarazaki et al. reported that the ALB level was negatively related to early death (within 48 h) among patients with AKI who were receiving CRRT due to sepsis and other diagnoses (*OR* = 0.52, 95% CI: 0.28–0.92) [23]. However, no recent study has addressed the association between ALB levels and death in sepsis patients with AKI undergoing CRRT alone. There are several reasons why the ALB level plays a crucial role in the prediction of endpoints among these patients. First, ALB is one of the most important proteins, accounting for 50% of the total proteins in human plasma. Sepsis is a severe systemic inflammatory response that leads to functional impairment of the vascular endothelial barrier and increased capillary permeability. Leakage of ALB into the interstitial space thus contributes to a decrease in plasma ALB concentration. Research has shown that the capacity of serum ALB in the interstitial space increases by 300% within hours of the onset of septic shock [24]. Second, the liver is the unique organ for ALB synthesis. Patients with sepsis have different degrees of ALB synthesis deficiencies because nutrients cannot be consumed and used efficiently in the liver. In addition, in the early stage of sepsis, the liver will prioritize the synthesis of acute phase proteins such as CRP over ALB. Third, sepsis patients have a hypermetabolic and hypercatabolic state, increasing the catabolism of ALB [25]. Fourth, serum ALB has various physiological functions, including anti-oxidation [26], anti-inflammation [27, 28] and maintenance of vascular endothelial function integrity [29], all of which play a role in reducing the adverse effects of inflammatory response and the incidence of organ failure [30–32]. The above findings suggest that the ALB level is independently and negatively associated with death at 28 days and 90 days among sepsis patients with AKI undergoing CRRT.

Our study had a number of advantages. First, we proved that there was a significant linear trend, namely the dose-effect relationship between ALB tertiles and the endpoints (*P* for trend < 0.05). Second, to explore the association between ALB levels and endpoints, we detect possible nonlinear correlation and threshold effect as well, which helped us study the correlation more accurately. Third, we used the MI method to evaluate the impact of missing data. The results of MI showed that the data were missing at random and did not result in significant bias. Fourth, the subgroup analysis and test for interaction indicated that the negative correlation between ALB levels and the endpoints was stable and significant interaction was found.

We recognized some limitations of our study as well. First, this was a retrospective cohort study which limits the generalization of its findings as a result of the retrospective nature of the study. Compared with prospective studies, it was more difficult to avoid exposure suspicion bias and other biases. Thus, further prospective studies are needed in this concern. Second, the present study included only the South Korean population; therefore, the conclusion cannot be extrapolated to other populations at present. Third, as with any observational study, there is an unavoidable potential for residual confounding. However, based on E-value computations, changes to our results from unmeasured confounding would be unlikely (E-value = 1.74 for death at 28 days and E-value = 1.79 for death at 90 days).

## Conclusion

In the South Korean population, the ALB level was independently negatively correlated with mortality in sepsis patients with AKI undergoing CRRT. A 1 g/dL increase in the ALB level was associated with a 25 and 27% lower risk of death at 28 days and 90 days, respectively. However, this conclusion needs to be taken with caution as this study has several limitations.

## Supplementary Information

**Additional file 1: Table S1.**

**Additional file 2: Table S2.**

**Additional file 3: Table S3.**

**Additional file 4: Table S4.**

## Abbreviations

ALB: Albumin
AKI: acute kidney injury
CRRT: continuous renal replacement therapy
HR: hazard ratio
CI: confidence interval
AKIN: Acute Kidney Injury Network
UO: urine output
CKD: chronic kidney disease
BMI: body mass index
SBP: systolic blood pressure
DBP: diastolic blood pressure
MAP: mean arterial pressure
aCCI: age-adjusted Charlson comorbidity index
MV: mechanical ventilation
HGB: hemoglobin
WBC: white blood cell
CRP: C-reactive protein
Cr: creatinine
BUN: blood urea nitrogen
GFR: Glomerular Filtration Rate
HCO_3_: bicarbonate
K: potassium
P: phosphate
SOFA: Sequential Organ Failure Assessment
APACHE II: Acute Physiology and Chronic Health Evaluation
SD: standard deviation
Q: quartile
K–M: Kaplan–Meier
STROBE: Strengthening the Reporting of Observational studies in Epidemiology
MI: multiple imputation
GAM: generalized additive model
N: number
